# On the strong-CP problem and its axion solution in torsionful theories

**DOI:** 10.1140/epjc/s10052-018-5972-0

**Published:** 2018-06-11

**Authors:** Georgios K. Karananas

**Affiliations:** Arnold Sommerfeld Center, Ludwig-Maximilians-Universität München, Theresienstraße 37, 80333 Munich, Germany

## Abstract

Gravitational effects may interfere with the axion solution to the strong-CP problem. We point out that gravity can potentially provide a protection mechanism against itself, in the form of an additional axion-like field associated with torsion.

The effective theory describing the dynamics of the QCD axion *a* contains a nontrivial interaction between the pseudoscalar and the QCD Chern–Simons topological density, and has the following schematic form 1

1 $$\begin{aligned} \mathscr {L}_a=\frac{1}{2}\partial _\mu a \partial ^\mu a +\frac{a}{f} G\widetilde{G}, \end{aligned}$$

with *f* the axion decay constant. We have used the shorthand notation

2 $$\begin{aligned} G\widetilde{G}= \frac{1}{2} \epsilon ^{\kappa \lambda \mu \nu }\mathrm {Tr}\left(G_{\kappa \lambda }{G}_{\mu \nu }\right), \end{aligned}$$

where $\epsilon _{\kappa \lambda \mu \nu }$ is the totally antisymmetric symbol, and $G_{\mu \nu }$ the QCD field strength.

It is clear that (1) is capable of solving the strong-CP problem, for the minimum of the axion potential forces the vacuum expectation value of $G\widetilde{G}$ to vanish. This in turn implies that physics does not depend on the CP-violating $\theta _\mathrm{QCD}$ parameter. It should be stressed that this holds true for the plethora of theories in which this specific coupling of *a* to the QCD Chern–Simons term appears, independently of their origin.^2^

It is quite illuminating to show why this is the case by using the dual formulation of QCD discussed thoroughly in [2] and later in [3, 4]. In this language, the vacuum superselection problem – or in other words the dependence of physics on $\theta _\mathrm{QCD}$ – translates into the presence of a long-range constant field associated with the three-form

3 $$\begin{aligned} \mathcal G_{\mu \nu \lambda }=\mathrm {Tr}\left(A_{[\mu }\partial _\nu A_{\lambda ]}+\frac{2}{3}A_{[\mu } A_\nu A_{\lambda ]}\right), \end{aligned}$$

where $A_\mu$ is the *SU*(3) gauge field, and the brackets $[\ldots ]$ denote antisymmetrization.

In the absence of the axion (as well as massless quarks), the topological vacuum susceptibility is nonzero [5]

4 $$\begin{aligned} \lim _{k\rightarrow 0}\int d^4x e^{ikx} \langle E(x)E(0)\rangle \ne 0, \end{aligned}$$

where we introduced $E\equiv \epsilon ^{\kappa \lambda \mu \nu }\partial _{\kappa } \mathcal G_{\lambda \mu \nu }$. At energies below the QCD confinement scale $\Lambda _\mathrm{QCD}$, $\mathcal G_{\kappa \lambda \mu }$ behaves as a massless field [6], since from (4) it follows that its propagator has a pole at vanishing virtuality. Its dynamics is captured by an effective lagrangian, whose (vacuum) equations of motion dictate that $E=\text {const.}$, in units of $\Lambda _\mathrm{QCD}$ [4]. This means that the theory possesses an infinite number of distinct vacua, one for each value of *E*.

On the other hand, when the axion is present, then in the dual picture it is replaced by a two-form $\mathcal A_{\mu \nu }=-\mathcal A_{\nu \mu }$, whose role is to put the massless field (3) in a Higgs phase by providing a (gauge-invariant) mass term for it.^3^ The low-energy dynamics of $\mathcal G_{\kappa \lambda \mu }$ is described by [2, 3]

5 $$\begin{aligned} \mathscr {L} =\frac{E^2}{\Lambda _{QCD}^4} +\frac{1}{f^2} \left( \mathcal G_{\kappa \lambda \mu }-\partial _{[\kappa }\mathcal A_{\lambda \mu ]}\right) ^2. \end{aligned}$$

The fact that the theory has now become gapped, means that the Chern–Simons field is now screened. This results into the vacuum susceptibility being zero, so the physics is independent of $\theta _\mathrm{QCD}$ and the strong-CP problem is solved.^4^

On general grounds, however, it is believed that gravity violates global symmetries, the aftermath of which might be the reintroduction of the strong-CP problem. This can be easily understood, since, in principle, extra terms – on top of the ones in (1) – can be generated by gravitational effects. This would result into the axion potential be displaced from the point where $\langle G\widetilde{G}\rangle =0$. In the absence of a theory of quantum gravity, it seems that there is no way of knowing the exact form of these contributions.

As realized in [2], the treatment of the problem in the dual description is particularly suggestive, for it makes clear that there is a unique way that the axion solution can be affected. This would correspond to the presence of an additional three-form field of gravitational origin with a massless pole in its propagator, which also couples with $\mathcal A_{\mu \nu }$.

Simply by counting the degrees of freedom in the theory, we notice that the number of the three-forms in this case would exceed the number of axions. Thus, necessarily, one of the fields – or better say, one combination of the fields – will be in a Coulomb phase.

It turns out that the suitable gravitational candidate is the following three-form

7 $$\begin{aligned} \mathcal R_{\mu \nu \lambda }= \Gamma ^\alpha _{\beta [\mu }\partial _{\nu }\Gamma ^\beta _{\lambda ]\alpha }+\frac{2}{3}\Gamma ^\alpha _{\beta [\mu }\Gamma ^\beta _{\nu |\gamma } \Gamma ^\gamma _{|\lambda ]\alpha }, \end{aligned}$$

with $\Gamma$ the Christoffel connection. Then, in complete analogy with QCD, there will be a nonvanishing “gravitational” vacuum susceptibility,

8 $$\begin{aligned} \lim _{k\rightarrow 0} \int d^4 x e^{ikx} \langle E'(x)E'(0)\rangle \ne 0, \end{aligned}$$

with $E'\equiv \epsilon ^{\kappa \lambda \mu \nu }\partial _\kappa \mathcal R_{\lambda \mu \nu }$. The above implies that the vacuum is also permeated by the constant field $E'\ne 0$; consequently, in the dual picture we find that the effective theory boils down to [2, 3] 5

9 $$\begin{aligned} \mathscr {L}= \frac{E^2}{\Lambda _{QCD}^4} +\frac{E'^{\,2}}{\Lambda ^4} +\frac{1}{f^2}\left(\alpha _G \mathcal G_{\mu \nu \lambda }+\alpha _R \mathcal R_{\mu \nu \lambda }-\partial _{[\mu }\mathcal A_{\nu \lambda ]}\right)^2, \end{aligned}$$

where $\Lambda$ is a scale set by the correlator (8), which need not necessarily be large, and $\alpha _G$, $\alpha _R$ constants. [As a side note, in the “conventional picture,” the aforementioned mixing between $\mathcal R_{\kappa \lambda \mu }$ and $\mathcal A_{\mu \nu }$, corresponds to (1) being supplemented by the term

11 $$\begin{aligned} \frac{a}{f}R\widetilde{R}, \end{aligned}$$

where

12 $$\begin{aligned} R\widetilde{R} = \frac{1}{2} \epsilon ^{\kappa \lambda \mu \nu }R^\rho _{\ \sigma \kappa \lambda }{R}^\sigma _{\ \rho \mu \nu }, \end{aligned}$$

is the gravitational parity-odd density. Here, $R^\kappa _{\ \lambda \mu \nu }$ is the Riemann curvature tensor.] We can go a step further and make explicit that the axion solution is affected. To this end, it is convenient to diagonalize the mass term in the above by introducing

13 $$\begin{aligned} g_{\mu \nu \rho }= & {} \alpha _G \mathcal G_{\mu \nu \rho }+\alpha _R \mathcal R_{\mu \nu \rho },\quad \text {and}\nonumber \nonumber \\ r_{\mu \nu \rho }= & {} \alpha _G \mathcal G_{\mu \nu \rho }-\alpha _R \mathcal R_{\mu \nu \rho }. \end{aligned}$$

Expressed in terms of these new fields, it is easy to see that only *g* gets a mass, while *r* remains massless. Given the previous discussion, this is something that should hardly come as a surprise.

A protection mechanism against the gravitational contribution is the existence of yet another two-form $\mathcal A'_{\mu \nu }$ in the theory, such that it screens the second field as well [2–4]. For instance, this can emerge from the presence of neutrinos in the theory, as was suggested in [3]. Various aspects of this proposal were further investigated and generalized in [7, 8].

Alternatively, $\mathcal A'_{\mu \nu }$ can be identified with an axion-like degree of freedom that couples to $G\widetilde{G}$, $R\widetilde{R}$, or both. It is tempting to entertain the possibility that this field actually be of gravitational origin. This means that gravity would have an inherent protection mechanism, which counterbalances its original effect on the strong-CP problem. Let us discuss how this can indeed be the case.

It has been known for many years that gauging the Poincaré group yields the Einstein–Cartan–Sciama–Kibble theory [9–12].^6^ In order to achieve invariance under local translations and Lorentz transformations, one needs more degrees of freedom than in General Relativity: the *a priori* independent vielbein and spin connection, whose respective field strengths are torsion and curvature.

It should be noted that it is in principle possible to eliminate the extra degrees of freedom by imposing vanishing torsion. In a four-dimensional spacetime this gives rise to twenty-four constraint equations that allow to express the connection in terms of the derivatives of the vielbein (or equivalently the metric).

If, on the other hand, torsion is not eliminated, then the presence of chiral fermions in this context has quite interesting implications. The fermionic (torsionful) covariant derivative involves the axial four-vector of the torsion;^7^ a rather nontrivial consequence of this interaction is the emergence of a pseudoscalar axion-like field $\varphi$, which couples derivatively with the spinorial axial current $j^\mu _5$ [20–24].^8^

However, it is well known that due to the chiral anomaly, the divergence of $j^\mu _5$ is nonzero. Consequently, $\varphi$ will interact (in a classically shift-symmetric manner) with the Chern–Simons topological densities associated with QCD and gravity. It should stressed at this point that the latter mixing appears obiquitously in the context of torsionful theories, so *its presence need not be assumed* (for instance see [20–26]). Let us note in passing that the divergence of the current might comprise other terms too, such as the *U*(1) as well as *SU*(2) CP-odd invariants which, nevertheless, are irrelevant for the present discussion, so we have tacitly ignored them.

In the dual picture, the presence of $\varphi$ with these “special” couplings to $G\widetilde{G}$ and $R\widetilde{R}$, translates into the effective theory (10) becoming

14 $$\begin{aligned} \mathscr {L}&= \frac{E^2}{\Lambda _{QCD}^4} +\frac{E'^{\,2}}{\Lambda ^4} +\frac{1}{f^2}\left(\alpha _G \mathcal G_{\mu \nu \lambda }+\alpha _R \mathcal R_{\mu \nu \lambda }-\partial _{[\mu }\mathcal A_{\nu \lambda ]}\right)^2 \nonumber \\&\quad +\frac{1}{f'^{\,2}}\left(\beta _G \mathcal G_{\mu \nu \lambda }+\beta _R \mathcal R_{\mu \nu \lambda }-\partial _{[\mu }\mathcal A'_{\,\nu \lambda ]}\right)^2 \ . \end{aligned}$$

Here, $\beta _G$ and $\beta _R$ are constants, while $f'$ is the decay constant of $\varphi$, which is not a free parameter and its value is roughly of the order of the Planck scale [20–24]. We notice that, as long as $\alpha _G/\alpha _R\ne \beta _G/\beta _R$,^9^ both the QCD as well as the gravitational three-forms have entered a Higgs phase, so there are no long-range fields in the vacuum and the solution to the strong-CP problem persists.

## Footnotes

1 Throughout this article we will not keep track of irrelevant numerical factors.

2 For example, in the original Peccei–Quinn proposal [1], the axion emerges as the pseudo-Goldstone boson of a spontaneously broken anomalous *U*(1) symmetry.

3 In the dual formulation, there is the gauge invariance

6 $$\begin{aligned} \mathcal G_{\kappa \lambda \mu }\rightarrow \mathcal G_{\kappa \lambda \mu }+\partial _{[\kappa } c_{\lambda \mu ]} \ ,\quad \mathcal A_{\lambda \mu }\rightarrow \mathcal A_{\lambda \mu }+c_{[\lambda \mu ]}, \end{aligned}$$

which obviously cannot be broken.

4 This can also be achieved with massless quarks.

5 Note that, on top of (5), the lagrangian (8) is also invariant under the dual version of diffeomorphisms,

10 $$\begin{aligned} \mathcal R_{\kappa \lambda \mu } \rightarrow \mathcal R_{\kappa \lambda \mu } + \partial _{[\kappa }d_{\lambda \mu ]},\quad \mathcal A_{\lambda \mu } \rightarrow \mathcal A_{\lambda \mu } +d_{[\lambda \mu ]}. \end{aligned}$$

6 The Poincaré group can be gauged, for example, by employing the Callan–Coleman–Wess–Zumino coset construction [13, 14], for the case of spacetime symmetries [15, 16]. See also [17–19] and references therein for a number of generalizations and applications.

7 Torsion can be decomposed under the Lorentz group into a vector, an axial vector and a tensor with mixed symmetries.

8 Contrary to [20–24], however, *we do not identify* this field with the standard Peccei–Quinn axion.

9 It would be somehow peculiar for this relation to not be true in general, since $\alpha _G$ and $a_R$ are model-dependent parameters.
